# Retrospective ACEs predict complex PTSD symptoms in a large sample of Chinese young adults longitudinally: the moderating role of self-compassion

**DOI:** 10.1186/s12888-024-05830-z

**Published:** 2024-06-06

**Authors:** Yizhen Ren, Shuhan Yang, Yu Peng, Aiyi Liu, Zibin Zhu

**Affiliations:** 1 Faculty of Psychology, Beijing Normal University, Beijing, 100875 China; 2 Faculty of Education, Yunnan Normal University, Kunming, 650500 China; 3 Students Mental Health Education & Counseling Center, Kunming University of Science and Technology, Kunming, 650500 China; 4 Faculty of Social Sciences & Liberal Arts, UCSI University, Kuala Lumpur, 56000 Malaysia; 5 School of Philosophy, Psychology and Language Science, University of Edinburgh, Edinburgh, UK

**Keywords:** Adverse childhood experiences, CPTSD, Multidimensional self-compassion

## Abstract

This longitudinal study in Mainland China (2021–2022) explored the impact of adverse childhood experiences (ACEs) on complex posttraumatic stress disorder (CPTSD) symptoms, with a focus on the role of self-compassion. Among 18,933 surveyed university students, 21.2% reported experiencing at least one ACE. Results revealed a clear relationship between ACEs and CPTSD symptoms. Furthermore, self-compassion, particularly the dimensions of self-judgment and isolation, moderated the association between retrospective ACEs and posttraumatic stress disorder (PTSD) and disturbance in self-organization (DSO) symptoms. These findings highlight the enduring impact of ACEs on CPTSD symptoms and emphasize the importance of early identification and targeted interventions, especially addressing self-judgment and isolation, to mitigate CPTSD risk among young Chinese adults.

## Introduction

As a distinct construct different from PTSD, CPTSD has been proposed as potentially arising after enduring extreme, prolonged, or repetitive events perceived as immensely threatening or horrifying, with limited chances of escape. These events, termed “complex traumas,” include instances such as childhood physical or sexual abuse, war, genocide campaigns, and torture [1]. The PTSD diagnosis in ICD-11 focuses on three core elements: flashback, avoidance and hypervigilance [2–4]. The diagnosis of CPTSD in ICD-11 requires the simultaneous presence of these three core elements, along with three additional symptom domains representing DSO: affect dysregulation, negative self-concept, and interpersonal difficulties [4–6]. DSO means the disturbance in self-organization [5]. Notably, affect dysregulation signifies problems in affect regulation, negative self-concept denotes a negative cognition of oneself, and interpersonal difficulties indicate disturbances in relationships [4]. In contrast to PTSD, CPTSD demonstrates a stronger association with interpersonal trauma, particularly childhood maltreatment [7, 8]. Symptoms of CPTSD are believed to stem from a complex combination of childhood traumas, with adverse childhood experiences (ACEs) representing an accumulation of trauma over an extended period [9]. This accumulation, as highlighted by the cascade model of CPTSD symptoms [8, 9], contributes to increased risks of CPTSD symptoms [6, 8, 9].

Given the prevalence of ACEs in China [10–13], this study employs a longitudinal design to explore the connection between retrospective ACEs and CPTSD symptoms among Chinese young adults. Young adulthood is a critical phase characterized by the highest incidence of mental health problems and significant developmental changes for individuals [14, 15]. The adverse effects of ACEs may have enduring consequences [16], as individuals with a history of adverse childhood experiences tend to exhibit more stable patterns of mental health problems, such as severe substance use, over time [15]. Furthermore, the risk of mental health problems associated with ACEs, such as depression, tends to increase over time [17]. Hence, this study utilized a longitudinal design to explore the relationship between retrospective ACEs and CPTSD symptoms in Chinese young adults. Additionally, recognizing that individuals respond differently to accumulative ACEs in terms of CPTSD symptom severity, the study delves into the potential moderating role of self-compassion dimensions in this relationship. Self-compassion, reflecting one’s self-attitude during times of suffering [18–20], could potentially influence the development of psychopathology [21, 22]. Consequently, this study incorporates a longitudinal approach to investigate how self-compassion may moderate the link between accumulative ACEs and CPTSD symptoms.

### Retrospective ACEs predict CPTSD symptoms longitudinally

Recent research has offered evidence of the high prevalence of childhood adversity in China [10–13]. Studies have shown that approximately half of Chinese young adults have reported experiencing at least one ACE [6], and a significant proportion, around one in three or two individuals, have reported exposure to two or more ACEs [6, 23]. The ACEs encompass various forms of abuse, including sexual, physical, or emotional abuse, as well as instances of neglect and adverse family circumstances, all of which can occur during childhood [24–28]. Overall, the ACEs include a broad range of events that constitute childhood trauma [29]. Extensive research conducted on ACEs has consistently shown similar findings. Childhood adversity is a common occurrence, individuals often experience multiple types of adversity rather than just one [4, 6, 30, 31]. The impact on the development of children exposed to multiple risk factors is more severe than that of single exposures [8, 32]. The nature, timing, and dosage of adverse experiences all play crucial roles [33]. Previous studies investigating the correlation between ACEs and depressive symptoms have shown that the likelihood of depression rises with a greater cumulative count of ACEs endured [26]. There is a robust dose-response relationship between ACE score and the lifetime and recent probability of depressive disorder [29]. The odds ratios for adult depression increase by 26.3% with one ACE, 86.9% with two ACEs, 130.0% with three ACEs, and 182.6% with four or more ACEs [34]. Furthermore, cumulative trauma exposure in childhood may serve as a fundamental risk factor for the early onset of developmental disorders, potentially leading to CPTSD symptoms [35]. Therefore, exposure to multiple risks has more detrimental effects on development [36]. To measure cumulative risk, the concept of an “ACE score” has been introduced, which represents the total number of ACE categories experienced [30, 37]. The ACE score has significantly enhanced our understanding of the pervasive and harmful effects of multiple forms of early childhood adversity. The accumulation of ACEs can have detrimental effects, including depression [26, 29] and CPTSD symptoms [4, 6, 8]. Particularly when exposed to sustained or repeated traumas during childhood, individuals may exhibit a symptom profile that extends beyond typical posttraumatic stress symptoms, involving disturbances in affective and interpersonal self-regulation [4, 6, 8, 38, 39], as well as disruptions in self-concepts [38]. This aligns with the concept of CPTSD [4, 6].

Recent research has proposed the cascade model of CPTSD symptoms [9], according to which the accumulation of ACEs holds the potential to initiate a sequence of socio-interpersonal factors. These may manifest as dysfunctional interpersonal bonding behaviors, subsequently exerting influence over a series of cascading steps [9]. These steps involve multiple interactional processes that include various social entities, such as partners, family members, and friends of the survivors. These interactions wield the capacity to either nurture or impede the development of symptoms associated with CPTSD [9]. Hence, exposing to prolonged and repeated ACEs, individuals are particularly likely to develop CPTSD symptoms overall [8]. Additionally, other theories of psychopathology also explain the linkages between ACEs and CPTSD symptoms. For instance, the vulnerability-stress model suggests that major negative events like ACEs increase vulnerability to mental health difficulties [40]. ACEs occurring during sensitive periods of development, when children lack sufficient coping resources, can lead to the development of pervasive toxic stress and heightened vigilance in later life [41]. Previous research has also found a dose-response relationship between cumulative trauma and the severity and complexity of psychological symptomatology [42], suggesting that a higher number of ACEs may lead to more severe CPTSD symptoms [8]. Based on these findings, the present study hypothesized a direct impact of ACEs on CPTSD symptoms. Importantly, the period of adolescence and young adulthood is critical for personality development and the acquisition of self-regulation capacities [43], which may increase susceptibility to developing or prolonging CPTSD symptoms [8]. Therefore, it is crucial to identify modifiable buffering factors and potential mechanisms associated with CPTSD in this population and allocate greater attention to these matters.

### The moderating role of multidimensional self-compassion

As previously mentioned, CPTSD can arise from exposure to an event or a series of events that are highly distressing, prolonged, or repetitive, making it difficult or impossible to escape [4, 8, 44]. However, it is important to note that not all individuals who experience repeated traumatic events will necessarily exhibit symptoms of PTSD or CPTSD. Research studies [45–48] have shown that various factors can influence the outcome. For instance, individuals with a history of high betrayal traumas who possess a dialectical self-view may experience enhanced adjustment and reduced PTSD symptoms due to a decrease in the negative impact of betrayal traumas on self-compassion [45]. Conversely, some individuals may experience a worsening of PTSD symptoms over time, especially those who heavily rely on avoidant coping strategies and are highly reactive to trauma reminders [48]. In short, there are other important factors that may moderate the relationships between ACEs and CPTSD symptoms. Self-compassion, a significant factor influencing trauma-related symptoms, reflects individuals’ attitudes toward themselves during times of suffering. It has garnered increasing attention from researchers as a self-attitude concept [18, 19, 49]. It refers to people’ s tendency to understand, forgive, and avoid self-judgment when facing frustration [18, 19, 49]. Neff [19] defined self-compassion using three dimensions: (a) self-kindness and self-judgment, (b) common humanity and isolation, and (c) mindfulness and overidentification. Typically, self-compassion is measured by calculating the total score of the self-compassion scale [50–52], while reversing items related to self-judgment, isolation, and over-identification. Prior research has also suggested the moderating role of self-compassion in influencing psychological adjustment outcomes [21, 22]. For example, Keng et al [21] demonstrated that self-compassion moderated the link between perceived COVID-19 health risk and depressive symptoms, as well as the relationship between perceived impact on daily life and anxiety symptoms. An additional study [22] emphasizes the significance of self-compassion as a strong factor in resilience, which is associated with decreased psychopathology. This study also highlights its role in moderating the connection between self-criticism, a powerful risk factor and psychopathology within the context of child maltreatment.

Notably, the studies mentioned above [21, 22] have only used the total score self-compassion in influencing psychological outcomes. However, there is controversy regarding the reliance on the total score as an indicator of self-compassion. The different dimensions are relatively independent [18, 53, 54] and have distinct effects on individuals’ mental health [55, 56]. Previous research has consistently demonstrated that different dimensions of self-compassion exert distinct influences on mental health outcomes, including depression [8, 56, 57]. Additionally, self-kindness, self-judgment, mindfulness, and isolation subscales have been found to be significant predictors of mental health problems like depression, while common humanity and overidentification did not significantly predict depressive symptoms [58]. Individuals with low levels of compassion and self-kindness tend to experience poor psychological adjustment, with isolation being particularly associated with negative emotions [59]. Recent studies have further highlighted that the positive dimension of self-compassion can alleviate mental health problems, whereas the negative dimension may exacerbate issues such as depression and nonsuicidal self-injury [55, 56]. Given this background, our hypothesis suggests that self-judgment, isolation, and overidentification moderate the relationship between ACE and CPTSD symptoms.

### The present study

To our knowledge, only a few studies have assessed the dose-response relationship between retrospective ACEs and CPTSD symptoms in mainland China [6, 8]. However, these studies have certain limitations. Firstly, they used relatively small sample sizes (less than 3000). Secondly, they relied on a cross-sectional study design instead of adopting a longitudinal approach to examine the associations [6, 8]. Thirdly, these studies did not consider individual moderating mechanisms in the longitudinal relationships between retrospective ACEs and CPTSD symptoms. Based on this background, the present study aimed to examine the longitudinal predictive effects of retrospective ACEs on CPTSD symptoms in Chinese young adults. Additionally, the study aimed to explore the moderating role of multidimensional self-compassion in these associations. We hypothesized that retrospective ACEs could positively predict CPTSD symptoms longitudinally. Additionally, self-judgment, isolation, and overidentification moderated the relationship between ACE and CPTSD symptoms.

## Methods

### Procedure

The research was carried out at a university located in Yunnan Province, China. This study is part of a longitudinal research project with a one-year gap between two phases of data collection. The first assessment was conducted from September to October 2021, and the second follow-up survey took place in September 2022, maintaining a one-year interval. Both surveys were administered online, with participants receiving a thorough introduction to the study’s objectives, their rights as participants, and any potential risks involved beforehand. In the first phase of measurement, university students were invited to participate in the study. They were requested to fill out surveys regarding socio-demographic information and past ACEs after giving informed consent. One year later, the same cohort of participants was invited to report their self-compassion levels and CPTSD symptoms. The study obtained approval from the Institutional Review Board of the author’s affiliated institution. Participants were granted the autonomy to withdraw from the study at any point.

### Participants

In the first survey, a total of 33,797 university students participated in the study. In the current study, if any of the questionnaire items were left unanswered, the survey was considered incomplete. Incomplete questionnaires were not included in the analysis. Participants with incorrect student numbers (e.g., digits not matching the correct student number) were considered invalid. To maintain data quality, response options demanding fixed answers (e.g., “Please choose ‘4’”) were strategically included at various points in the survey. Any incomplete or unsubmitted surveys were excluded from the analysis, encompassing those with invalid responses in the quality checking items. After excluding invalid and extreme value data, 28,202 young adults provided valid socio-demographic information (age range: 18–30 years, mean age 22.07 ± 2.04 years) and retrospective ACEs. In the subsequent follow-up survey conducted one year later, a total of 18,933 university students participated in the reassessment, providing data on their CPTSD symptoms and self-compassion levels. After excluding 14,927 participants who did not report any ACE, the final sample includes 4,006 university students who reported experiencing at least one ACE. The age of the final sample ranges from 18 to 30 years, with a mean age of 21.77 ± 1.91 years. Among this sample, 2,453 (58.8%) were males and 1,652 (41.2%) were females. Detailed demographic statistics can be found in Tables 1 and 2.

**Table 1:** The Demographic information of participants

Variables	T1 Following-up (*N* = 18,933)		T1 Missing (*N* = 9269)		χ^2^/t	*p*
	N/M	%/SD	N/M	%/SD		
**Age**	22.07	2.04	23.52	2.00	56.870	< 0.001
**Gender**					14.309	< 0.001
Male	11,948	63.1%	5634	60.8%		
Female	6985	36.9%	3635	39.2%		
**Education level**					63.584	< 0.001
Studying for a bachelor degree	12,825	67.7%	6711	72.4%		
Studying for a master degree	6108	32.3%	2558	27.6%		
**Subjective socio-economic status**					17.331	< 0.001
Worse	4562	24.1%	2439	26.3%		
Average	13,541	71.5%	6458	69.7%		
Better	830	4.4%	372	4.0%		

**Table 2:** The Demographic information of the final sample

Variables	Final sample(*N* = 4006)	
	N/M	%/SD
Age	21.77	1.91
Gender		
Male	2354	58.8%
Female	1652	41.2%
Education level		
Studying for a bachelor degree	3035	75.8%
Studying for a master degree	971	24.2%
Subjective socio-economic status		
Worse	1240	31.0%
Average	2591	64.7%
Better	175	4.4%

### Measures

#### Demographic variables

Participants were invited to report their age, gender (1 = male, 2 = female), subjective socioeconomic status (1 = lower, 2 = average, 3 = higher), and educational level (1 = undergraduate, 2 = graduate).

#### Adverse childhood experiences

The revised version of the Adverse Childhood Experiences Inventory was utilized to measure adverse childhood experiences occurring before the age of 18 [24, 60]. This inventory includes 10 items addressing physical abuse, emotional abuse, emotional neglect, sexual abuse, physical neglect, parental separation or divorce, familial substance abuse, domestic violence, familial mental illness, and familial incarceration [61]. These experiences were individually scored as 0 (“no”) or 1 (“yes”), and then cumulatively added to calculate the total ACEs score. A higher total score indicates a greater number of adverse experiences. The ACE inventory has been revised and validated among Chinese young adults [62].

#### Complex posttraumatic stress disorder (CPTSD) symptoms

In the second wave of measurement, CPTSD symptoms were assessed using the International Trauma Questionnaire [63], with the translated Chinese version of ITQ having previously demonstrated good psychometric properties as validated in earlier studies [4, 6, 8, 64]. The questionnaire included a total of 18 items, of which 12 items were used to measure the symptom score of CPTSD [63]. Six of these items assess PTSD symptom scores, such as “Having upsetting dreams that replay part of the experience or are clearly related to the experience?” Furthermore, the remaining six items measure DSO symptom scores, for instance, “When I am upset, it takes me a long time to calm down.” CPTSD shows six symptom clusters (i.e., flashback avoidance, hypervigilance, affect dysregulation, negative self-concept, and interpersonal difficulties). The rating scale ranged from 0 (“Not at all”) to 4 (“Extremely”). Higher total scores indicate more severe symptoms. The Cronbach α coefficient for this sample of CPTSD symptoms in this study was 0.909.

#### Self-compassion

Participants in Wave 2 were given the 12-item Self-Compassion Scale (SCS), which assesses six dimensions of self-compassion, including self-kindness, self-judgment, common humanity, isolation, mindfulness, and overidentification [19]. The items on the Short Form of the SCS (SCS-SF) all come from the original 26-item version. Responses were identified on a 5-point scale ranging from “Almost Never” to “Almost Always.” This scale has been validated [65] and used to measure self-compassion in Chinese populations [59]. In this study, the Cronbach α coefficient for self-compassion is 0.771.

### Data analyses

Descriptive statistics, including the means and standard deviations of the continuous variables, and Pearson correlational analysis, were conducted using SPSS 27.0. Structural equation modeling was employed to examine the moderating role of multidimensional self-compassion in the predicting effects of ACEs on CPTSD symptoms using Mplus 8.3. Prior to the analysis, ACEs scores, six self-compassion dimensions, and CPTSD symptoms were standardized to facilitate the interpretation of path coefficients in the results. Sociodemographic variables, including age, gender (1 = male and 2 = female), educational status (1 = Studying for a bachelor’s degree and 2 = Studying for a master’s degree), and subjective SES (1 = worse, 2 = average, and 3 = better) were controlled for in the analysis.

## Results

### Descriptive statistics

To assess potential differences between those who reported ACEs and those who did not, we used chi-square tests and t-tests to analyze disparities in participant characteristics. The results revealed significant differences between the two datasets in terms of age (*t* = 10.822, *p* < 0.001), gender (χ^2^ = 41.200, *p* < 0.001), education level (χ^2^ = 149.644, *p* < 0.001), and subjective socio-economic status (χ^2^ = 1 32.447, *p* < 0.001). Given the substantial sample size in our study, the p-values may not adequately reflect the true differences between the samples [66, 67]. Therefore, we proceeded with a more in-depth assessment of the effect sizes for variables that showed significant differences. The effect sizes observed between the two datasets were small for age (*t* = 10.822, *p* < 0.001, *d* = 0.26), gender (χ^2^ = 41.200, *p* < 0.001, Phi = 0.047), education level (χ^2^ = 149.644, *p* < 0.001, Phi = 0.089), and subjective socio-economic status (χ^2^ = 132.447, *p* < 0.001, Phi = 0.084). Based on the comprehensive results, we think that there is not a substantial disparity in the basic demographic information between who did or did not report any ACE.

After excluding the young adults who did not report an ACE, the final sample included 4006 young adults with ACEs. The sample consisted of 2354 (58.8%) males and 1652 (41.2%) females. Detailed demographics of the participants with ACEs are displayed in Table 1. In Chinese young adults, the mean number of ACEs participants reported was 1.43 (SD = 0.91), with 73.5% of participants (*N* = 4006) reporting experiencing a single ACE. The ACEs with the highest incidence were parental separation or divorce (38.8%). In the sample that reported experiencing at least one ACE (*N* = 4006), the prevalence of CPTSD is 1.77%, and the prevalence of PTSD is 1.37%. These findings align with previous studies [68]. In this study, the mean score of CPTSD symptoms was 4.80, with a standard deviation of 4.67. Moreover, the mean scores for flashback (M = 1.10, SD = 1.31), avoidance (M = 1.06, SD = 1.41), hypervigilance (M = 0.75, SD = 1.24), affect dysregulation (M = 1.47, SD = 1.39), negative self-concept (M = 1.28, SD = 1.64), and interpersonal difficulties (M = 1.02, SD = 1.48) were consistent with previous studies examining symptoms of CPTSD [6]. Prevalence rates of all ACEs and the average scores of CPTSD symptoms were reported in Table 3. The correlational information among the study variables are show in Table 4.

**Table 3:** Percentage of the college student cohort reporting multiple adverse childhood experiences (*N* = 4006)

Adverse childhood experiences	CPTSD symptoms				Number and percentage of students who have experienced the childhood adverse experience (%)		
	M (total score)	SD	M (item score)	SD	*N*		%
Physical abuse	5.56	5.54	0.46	0.46	543		13.55%
Emotional abuse	6.40	6.18	0.53	0.52	198		4.94%
Sexual abuse	5.68	5.66	0.47	0.47	274		6.84%
Emotional neglect	7.03	6.28	0.59	0.52	308		7.69%
Physical neglect	4.63	5.33	0.39	0.44	818		20.42%
Parental separation or divorce	3.74	4.45	0.31	0.37	1555		38.82%
Domestic violence	4.81	5.07	0.40	0.42	668		16.67%
Familial substance abuse	4.59	4.81	0.38	0.40	429		10.71%
Familial mental illness	3.82	4.62	0.32	0.38	616		15.38%
Familial incarceration	4.66	4.94	0.39	0.41	333		8.31%
Percentage of students with multiple adverse experiences	1		2		3	4	5
	73.54%		16.50%		5.87%	2.55%	0.80%
	6		7		8	9	10
	0.45%		0.15%		0.10%	0.02%	0.02%

**Table 4:** Intercorrelations, means, and standard deviations for study variables

Variable	1	2	3	4	5	6	7	8	9	10	11	12	13
1 Age	-												
2 Gender	-0.004	-											
3 education level	0.807^**^	0.060^**^	-										
4 subjective socio-economic status	-0.036^*^	0.118^**^	0.020	-									
5 Retrospective ACEs	-0.049^**^	0.024	-0.064^**^	-0.035^*^	-								
6 Self-kindness	0.012	-0.014	0.023	-0.003	-0.083^**^	-							
7 Self-judgment	-0.055^**^	-0.101^**^	-0.390^*^	0.017	0.081^**^	-0.176^**^	-						
8 Common humanity	-0.002	-0.021	0.390^*^	0.008	-0.070^**^	0.595^**^	-0.062^**^	-					
9 Isolation	-0.035^*^	0.001	-0.015	-0.003	0.114^**^	-0.134^**^	0.424^**^	-0.073^**^	-				
10 Mindfulness	0.018	-0.098^**^	0.018	-0.027	-0.069^**^	0.705^**^	-0.157^**^	0.568^**^	-0.161^**^	-			
11 Overidentification	-0.054^**^	0.006	.-0.051^**^	0.015	0.091^**^	-0.034^*^	0.452^**^	0.008	0.581^**^	-0.106^**^	-		
12 PTSD symptoms	-0.019	0.032^*^	-0.024	0.018	0.188^**^	-0.290^**^	0.277^**^	-0.201^**^	0.347^**^	-0.274^**^	0.306^**^	-	
13 DSO symptoms	-0.021	0.037^*^	-0.012	-0.006	0.151^**^	-0.313^**^	0.298^**^	-0.227^**^	0.374^**^	-0.320^**^	0.315^**^	0.587^**^	-
M					1.433	3.621	2.512	3.501	2.761	3.732	3.084	0.961	0.195
SD					0.906	0.788	0.900	0.813	0.937	0.796	0.829	0.707	0.286

*Note* Gender: 1 = males, 2 = females;

*p < 0.05; **p < 0.01; ***p < 0.001

### The predicting effects of retrospective ACEs on CPTSD symptoms

We initially conducted a regression model to assess the predictive effects of ACEs on CPTSD symptoms, encompassing self-compassion dimensions, without moderators and interaction terms. The outcomes of the baseline simple model are presented in Table 5. After accounting for age, gender, educational status, and SES, the simple regression model showed acceptable model fit: (χ^2^ = 351.04, *df* = 52, χ^2^/*df* = 6.75, RMSEA = 0.038, CFI = 0.978, TLI = 0.966, SRMR = 0.014). We observed a significant longitudinal dose-response relationship between the number of ACEs and CPTSD symptoms. Retrospective ACEs could significantly predict the level of PTSD (*β* = 0.103, *p <* 0.001) and DSO symptoms (*β* = 0.100, *p <* 0.001).

**Table 5:** The predicting effects of ACEs and self-compassion on CPTSD symptoms without interaction terms

Outcome: PTSD symptoms	Estimate	*P*	Outcome: DSO symptoms	Estimate	*P*
Age	0.008	0.429	Age	0.008	0.406
Gender	0.045	0.064	Gender	**0.058**	**0.010**
Educational level	-0.026	0.569	Educational level	-0.078	0.068
SES	0.025	0.264	SES	0.006	0.772
**ACEs**	**0.103**	**< 0.001**	**ACEs**	**0.100**	**< 0.001**
**Self-kindness**	**-0.137**	**< 0.001**	**Self-kindness**	**-0.186**	**< 0.001**
**Self-judgment**	**0.072**	**< 0.001**	**Self-judgment**	**0.097**	**< 0.001**
**Common humanity**	**-0.031**	**0.039**	**Common humanity**	**-0.054**	**< 0.001**
**Isolation**	**0.150**	**< 0.001**	**Isolation**	**0.225**	**< 0.001**
**Mindfulness**	**-0.053**	**0.002**	**Mindfulness**	**-0.077**	**< 0.001**
**Overidentification**	**0.118**	**< 0.001**	**Overidentification**	**0.147**	**< 0.001**

*Note* Gender: 1 = female, 2 = male; Educational status: 1 = Studying for the bachelor’s degree, 2 = Studying for the Master’s degree, F SES: 1 = worse, 2 = average, 3 = better

### The moderating role of multidimensional self-compassion

After including controlling variables, in the longitudinal relationship between retrospective ACEs and PTSD symptoms, the interaction term between ACEs and self-judgment (*β* = 0.055, *p* < 0.05), as well as the interaction term between ACEs and isolation (*β* = 0.030, *p* = 0.032), were found to be significant (See Table 6). This suggests that self-judgment and isolation played moderating roles in the associations between retrospective ACEs and PTSD symptoms. In the longitudinal relationship between retrospective ACEs and DSO symptoms, the interaction terms between ACEs and self-judgment (*β* = 0.053, *p <* 0.001) was found to be significant. This indicates that self-judgment serves as a moderator in the predictive relationship between retrospective ACEs and DSO symptoms. In addition, the moderating effect of positive dimension of self-compassion on PTSD and DSO was not significant.

**Fig. 1:**
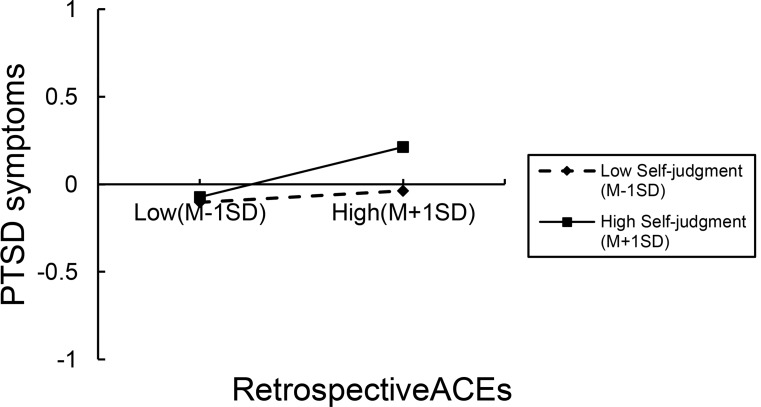
The Moderating Role of Self-Judgment Between Retrospective ACEs and PTSD Symptoms

Simple slope analysis (refer to Fig. 1) revealed that as self-judgment levels increased, the impact of ACEs on PTSD symptoms was magnified (see Fig. 1). When young adults demonstrated lower levels of self-judgment, ACEs was not significantly associated with PTSD symptoms (*β* = 0.033, *p* > 0.05). However, when young adults demonstrated higher levels of self-judgment, ACEs was significantly positively associated with PTSD symptoms (*β* = 0.187, *p* < 0.001).

**Fig. 2:**
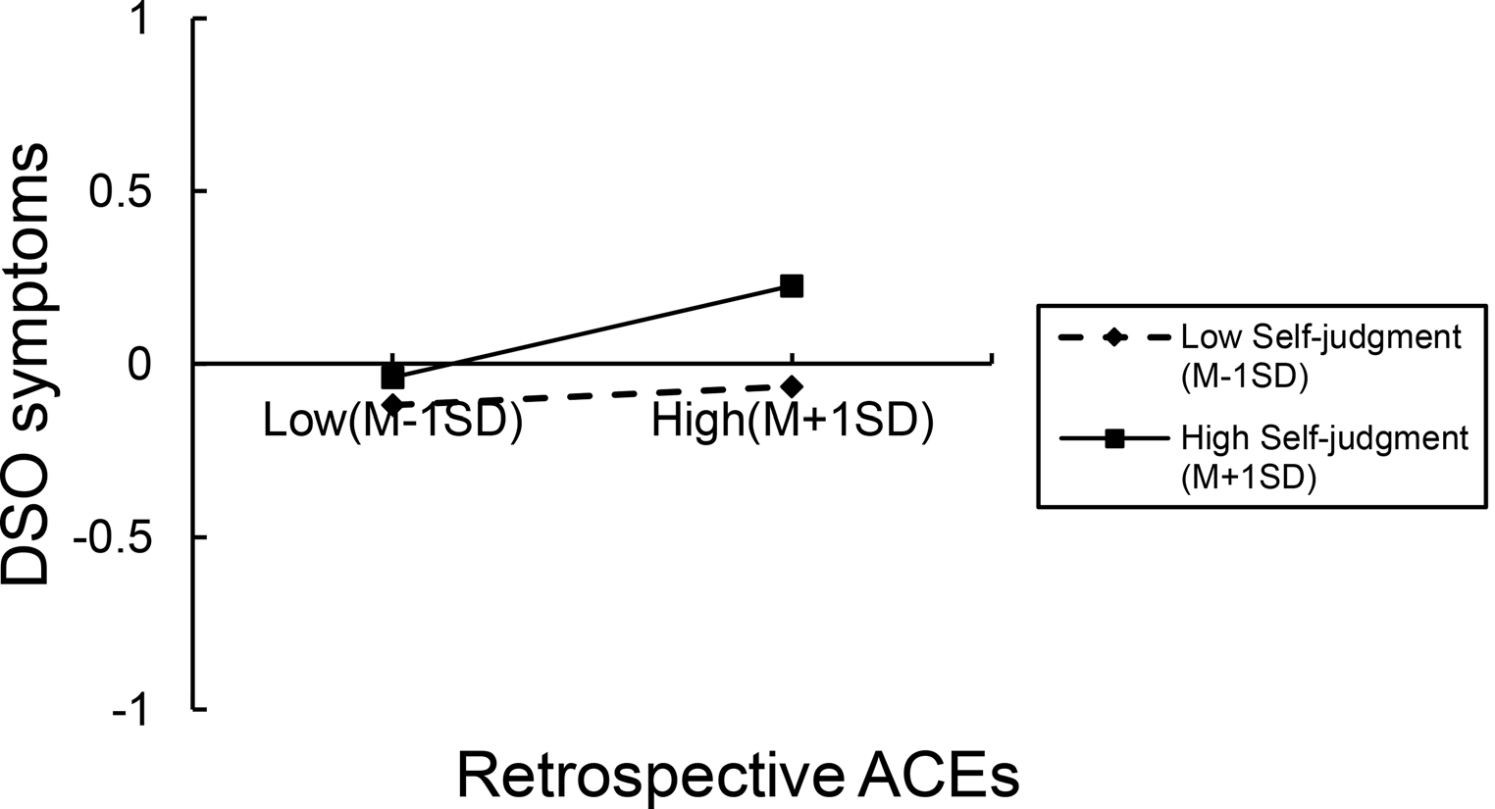
The Moderating Role of Self-Judgment Between Retrospective ACEs and DSO Symptoms

Simple slope analysis (refer to Fig. 2) indicated that as self-judgment levels increased, the impact of ACEs on DSO symptoms was amplified (see Fig. 2). When young adults demonstrated lower levels of self-judgment, ACEs were not significantly associated with DSO symptoms (*β* = 0.028, *p* = 0.094). However, when young adults demonstrated higher levels of self-judgment, ACEs was significantly positively associated with DSO symptoms (*β* = 0.165, *p* < 0.001).

**Fig. 3:**
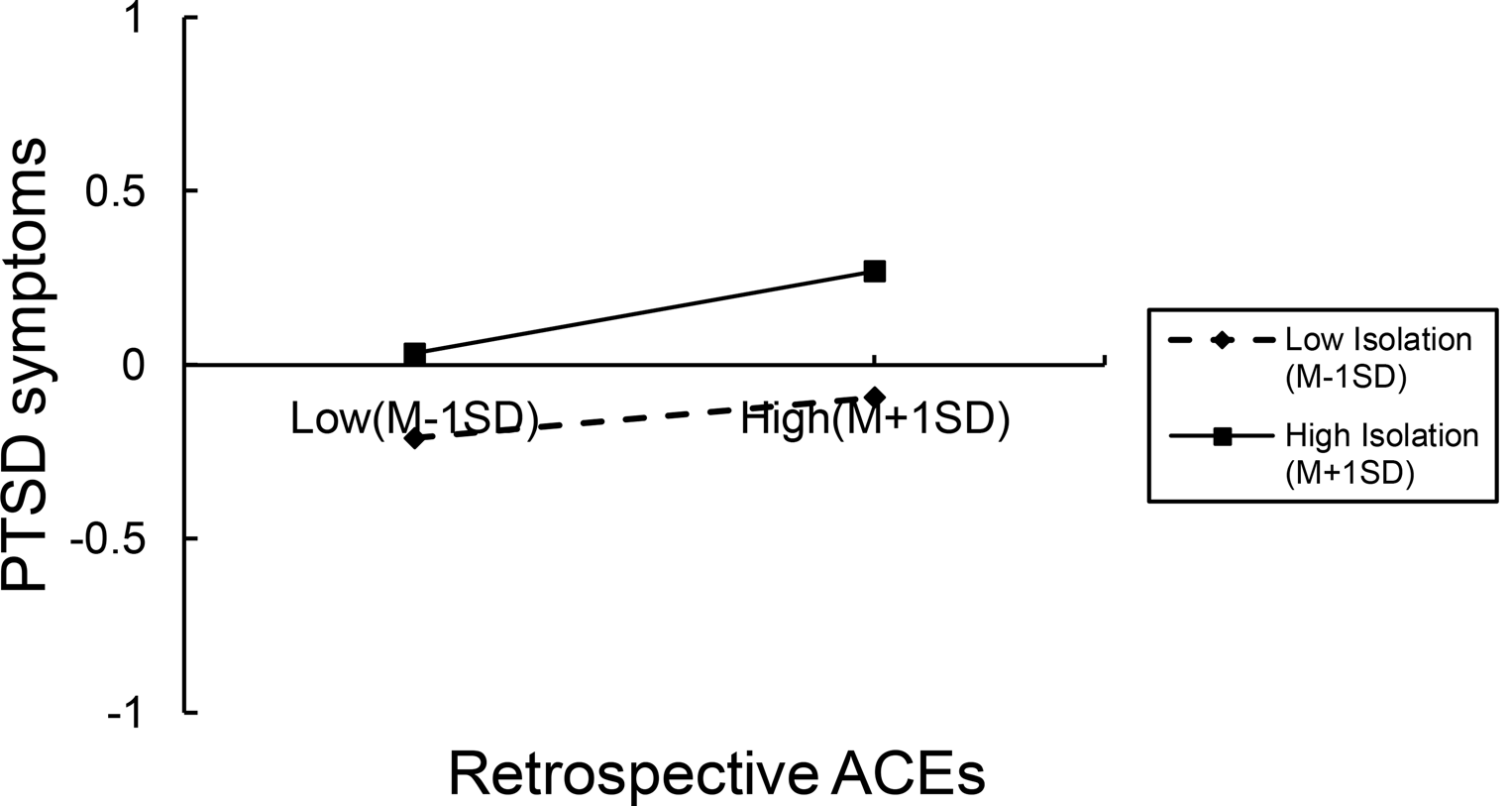
The Moderating Role of Isolation Between Retrospective ACEs and PTSD Symptoms

Simple slope analysis (see Fig. 3) demonstrated that as the level of isolation increased, the effect of ACEs on PTSD symptoms was enhanced (see Fig. 3). the positive link ACEs on PTSD symptoms was much stronger for high isolation (*β* = 0.118, *p* < 0.001) than low isolation (*β* = 0.059, *p* < 0.01).

**Table 6:** The predicting effects of ACEs and self-compassion on CPTSD symptoms after including controlling variables and interaction terms

Outcome: PTSD symptoms	Estimate	*P*	Outcome: DSO symptoms	Estimate	*P*
Age	0.008	0.422	Age	0.007	0.446
Gender	0.037	0.124	**Gender**	**0.049**	**0.029**
Educational level	-0.026	0.562	Educational level	-0.079	0.064
SES	0.026	0.228	SES	-0.006	0.757
ACEs	**0.088**	**< 0.001**	**ACEs**	**0.080**	**< 0.001**
Self-kindness	**-0.133**	**< 0.001**	**Self-kindness**	**-0.181**	**< 0.001**
Self-judgment	**0.070**	**< 0.001**	**Self-judgment**	**0.093**	**< 0.001**
Common humanity	**-0.030**	**0.042**	**Common humanity**	**-0.054**	**< 0.001**
Isolation	**0.152**	**< 0.001**	**Isolation**	**0.228**	**< 0.001**
Mindfulness	**-0.054**	**0.002**	**Mindfulness**	**-0.078**	**< 0.001**
Overidentification	**0.116**	**< 0.001**	**Overidentification**	**0.144**	**< 0.001**
ACEs × Self-kindness	-0.021	0.173	ACEs × Self-kindness	-0.018	0.212
ACEs × Self-judgment	**0.055**	**< 0.001**	**ACEs × Self-judgment**	**0.053**	**< 0.001**
ACEs × Common humanity	-0.009	0.538	ACEs × Common humanity	-0.026	0.067
ACEs × Isolation	**0.030**	**0.032**	ACEs × Isolation	0.007	0.595
ACEs × Mindfulness	-0.003	0.853	ACEs × Mindfulness	-0.008	0.556
ACEs × Overidentification	-0.021	0.155	ACEs × Overidentification	0.019	0.166

*Note* Gender: 1 = female, 2 = male; Educational status: 1 = Bachelor’s degree, 2 = Master’s degree, 3 = Doctor’s degree; Family type: 1 = Intactness, 2 = non-intactness; SES: 1 = worse, 2 = average, 3 = better

## Discussion

To the best of our knowledge, this study was the first to employ a longitudinal design to investigate the predictive effects of retrospective ACEs on CPTSD symptoms, along with exploring moderating mechanisms in these associations. This study revealed a relatively high rate of ACEs, indicating that ACEs were relatively common in Chinese young adults. In the current study, we found that retrospective ACEs could significantly predict the CPTSD symptom levels in Chinese young adults, reflecting a dose-response relationship between retrospective ACEs and CPTSD symptoms. Additionally, the moderating effects of self-judgment was identified in the relationship between retrospective ACEs and both PTSD and DSO symptoms. The dimension of isolation of self-compassion moderated the relations between retrospective ACEs and PTSD symptoms.

### The moderating role of self-compassion between ACEs and CPTSD symptoms

Similar to previous findings [8, 29, 42], our study revealed the existence of the dose-response relationship between ACEs and CPTSD symptoms, such that more accumulative ACE would lead to more severe CPTSD symptoms. In our current study, we have identified self-compassion as a moderator of the relationship between retrospective ACEs and CPTSD symptoms. This finding expands upon previous research that has shown the role of self-compassion in buffering the detrimental effects of traumatic experiences on trauma-related symptoms [22, 69]. While prior studies did not specifically examine the moderating effects of self-compassion in the associations between retrospective ACEs and CPTSD symptoms, earlier research has indicated that self-compassion can attenuate the effects of perceived stress on anxiety and depression prospectively [21, 70, 71]. Our findings underscore that self-compassion serves as an adaptive resource for coping with both traumatic experiences [22, 69] and distressing situations [21, 71]. Consequently, individuals who possess self-compassion are less likely to develop CPTSD symptoms in response to adverse experiences [8]. This highlights the potential significance of fostering self-compassion as a protective factor against the impact of trauma on mental health outcomes [72, 73]. This may be attributed to self-compassionate individuals being less likely to catastrophize, criticize themselves [73], and excessively identify with negative experiences [49, 59, 74]. During distressing times, they tend to cope by treating themselves with kindness and mindfully acknowledging negative thoughts without fixating on them [19, 73], enabling a balanced view of negative thoughts [74] and the use of more adaptive coping strategies [74, 75]. The findings highlight the value of cultivating self-compassion in coping with traumatic experiences, and provide support for the implementation of self-compassion programs for the public.

Furthermore, our study revealed that self-kindness plays a crucial role in the connection between ACE and CPTSD. Self-kindness can be considered as self-soothing behaviors associated with the mammalian contentment and safeness system (parasympathetic nervous system), which can downregulate the activated threat system based on Gilbert’s social mentality theory [76, 77]. Demonstrating self-kindness contributes significantly to an individual’s mental health and acts as a buffer against psychological distress [8, 78]. Recent research has underscored the role of self-kindness, emphasizing that being kinder to oneself can mitigate the negative effects of childhood adversity, facilitate healthier stress management, and reduce system-threatening activities, potentially alleviating CPTSD symptoms [8]. Other studies [79] have highlighted the specific and crucial role of self-kindness among the six dimensions of self-compassion in influencing PTSD-related outcomes. Young adults with higher levels of self-kindness tend to possess a better understanding of and care for themselves, addressing their inadequacies in a nonjudgmental manner [19, 49, 80–82]. Therefore, fostering self-kindness can help alleviate the negative emotions of young adults, enhance their understanding of traumatic experiences, and ultimately alleviate the symptoms of CPTSD.

### The specific moderating effect of self-judgment and isolation

This study delves into specific dimensions, revealing the pivotal role of self-judgment in moderating the link between past ACEs and PTSD symptoms, as well as the relationship between ACEs and DSO symptoms. Furthermore, the feeling of isolation also acts as a moderator in the connection between ACEs and PTSD symptoms. These findings support our hypothesis and are consistent with previous research [83, 84], underscoring the notable impact of isolation and self-judgment on personality disorder symptoms.

Our study demonstrates that the critical factor moderating posttraumatic psychological responses in young adults after ACEs isn’t primarily the absence of positive dimensions (self-kindness, common humanity, and mindfulness), but rather the presence of negative facets of self-compassion (self-judgment and isolation). This is in line with Rahmati’s study [84], where the dimensions of self-judgment and isolation emerged as the most potent predictors of personality disorder traits among the six self-compassion dimensions. Rahmati’s study [84] also highlights the dominant role of self-critique in individuals with personality disorder traits, further emphasizing the substantial moderating effects between retrospective ACEs and CPTSD symptoms in our current study. Specifically, prior research has emphasized the significant impact of isolation and self-judgment in the realm of borderline personality disorder symptoms [83, 84], paralleling similar aspects within CPTSD symptoms. These CPTSD symptoms encompass disruptions in affective and interpersonal self-regulation, as well as disturbances in self-concepts [4, 6, 8, 38, 39]. Furthermore, our study found no significant moderating effect of overidentification between ACEs and CPTSD symptoms. This finding is consistent with prior research [58, 85], where overidentification similarly did not significantly moderate the relationship between adverse experiences and negative mental health outcomes [85]. This may be attributed to the nature of CPTSD, which primarily revolves around interpersonal trauma [5]. Isolation and self-judgment are both intertwined with interpersonal factors, whereas overidentification primarily pertains to emotional aspects [19]. Given the observed deficiencies in various facets of self-compassion among those with CPTSD symptoms, fostering self-compassion could serve as an effective interventive strategy [8, 86]. This approach could particularly target self-critical perspectives, feelings of shame, self-worth issues, and negative self-schemas, ultimately alleviating CPTSD symptoms in individuals with ACEs [8, 86].

When young adults who have experienced ACEs become sensitive to external opinions and criticism, leading them to adopt self-critical attitudes and self-blame (self-judgment), and perceive themselves as solitary sufferers of pain (isolation), they become more susceptible to developing CPTSD symptoms. Our findings underscore that individuals with retrospective ACEs who tend toward self-blame and self-criticism (self-judgment), as well as a sense of isolated suffering (isolation), are at an elevated risk of experiencing severe CPTSD symptoms. Consistent with previous research [83, 84], our study highlights that the dimensions of self-judgment and isolation play a paramount role concerning symptoms with interpersonal implications. This significance of self-judgment and isolation in the context of personality disorders could potentially shed light on our current findings.

### Limitations

There are still some limitations to this study. First, it solely relied on self-reported questionnaires to assess CPTSD symptoms in Chinese young adults with ACEs. Future studies should consider incorporating multiple methods, such as clinical interviews, for a more comprehensive evaluation of CPTSD. Second, the study did not distinguish between different types of ACEs, which leaves us unaware of potential variations in the longitudinal effects of retrospective ACEs on CPTSD symptoms and the differing moderating roles of self-compassion dimensions. Third, the study did not measure or control for recent life events, which could potentially confound the results. To better understand the influence of retrospective ACEs on CPTSD symptoms, future research should control recent life event scores in the analysis. Fourth, this study solely investigated the moderating role of self-compassion, an individual factor, in the longitudinal associations between ACEs and CPTSD symptoms. It would be beneficial for future research to explore the relative roles of self-compassion compared to other individual and societal factors in buffering the effects of ACEs on CPTSD outcomes. Fifthly, in this study, 73.5% of participants (*N* = 4006) reported experiencing only one ACE. Therefore, the current sample may not be the most representative of cumulative ACEs. Finally, this study did not measure ACE and CPTSD repeatedly and simultaneously at multiple time points. In future studies, measuring ACE and CPTSD repeatedly will enhance the understanding of longitudinal relationships while accounting for concurrent correlations.

## Conclusions

This study revealed the longitudinal relationship between retrospective ACEs and CPTSD symptoms in Chinese young adults. Moreover, self-compassion prospectively buffers against the impact of retrospective ACEs on CPTSD symptoms. Self-judgment moderated the longitudinal links between retrospective ACEs and PTSD and DSO symptoms. Additionally, isolation moderated the prospective relationship between retrospective ACEs and PTSD symptoms. The study highlights that self-compassion acts as a buffer against the negative impact of retrospective ACEs on CPTSD symptoms, underscoring its importance in the link between retrospective ACEs and psychopathology. These results underscore the value of cultivating self-compassion and support the implementation of self-compassion programs for Chinese young adults with ACEs to cope with the effects of ACEs.

### Relevance for clinical practice

This study has some important clinical implications. First, it reveals a high prevalence of ACEs in Chinese young adults (21.2%), highlighting the need for special attention to this issue. Second, the study sheds light on the moderating roles of multidimensional self-compassion in the longitudinal links between retrospective ACEs and CPTSD symptoms. Therefore, clinical programs should pay particular attention to young adults who have experienced ACEs and focus on helping them develop a kinder way of treating themselves. This involves interventions that particularly focus on decreasing the levels of self-judgment and isolation. Third, considering individuals may have varying symptoms, intervention programs should tailor their approaches accordingly. For those experiencing severe DSO symptoms, programs should specifically focus on reducing the level of self- judgment.

## Data Availability

The datasets generated during and/or analyzed during the current study are available upon reasonable request from the corresponding author.
